# Exploration of Potential Biomarker Genes and Pathways in Kawasaki Disease: An Integrated *in-Silico* Approach

**DOI:** 10.3389/fgene.2022.849834

**Published:** 2022-05-09

**Authors:** Priyanka Srivastava, Chitra Bamba, Rakesh Kumar Pilania, Anu Kumari, Rajni Kumrah, Archan Sil, Surjit Singh

**Affiliations:** ^1^ Genetic Metabolic Unit, Department of Pediatrics, Advanced Pediatric Centre, Post Graduate Institute of Medical Education & Research, Chandigarh, India; ^2^ Allergy Immunology Unit, Department of Pediatrics, Advanced Pediatric Centre, Post Graduate Institute of Medical Education & Research, Chandigarh, India

**Keywords:** bioinformatics, biomarkers, hub genes, in-silico analysis, Kawasaki disease, microarray, transcriptomics analysis

## Abstract

Kawasaki disease (KD) is a common childhood systemic vasculitis with a special predilection for coronary arteries. Even after more than five decades of the initial description of the disease, the etiology of KD remains an enigma. This transcriptome data re-analysis study aimed to elucidate the underlying pathogenesis of KD using a bioinformatic approach to identify differentially expressed genes (DEGs) to delineate common pathways involved in KD. Array datasets from the Gene Expression Omnibus database were extracted and subjected to comparative meta-analysis for the identification of prominent DEGs. Fifteen hub genes with high connectivity were selected from these DEGs (*IL1B, ITGAM, TLR2, CXCL8, SPI1, S100A12, MMP9, PRF1, TLR8, TREM1, CD44, UBB, FCER1G, IL7R, and FCGR1A*). Of these 15 genes, five genes (*CXCL8, FCGR1A, IL1B, TLR2, and TLR8*) were found to be involved in neutrophil degranulation. To gain further insight into the molecular mechanism, a protein–protein network was established. Significantly enriched pathways based on the above-mentioned genes were mainly centered on biological regulation and signaling events. In addition, the pathway analysis also indicated that the majority of the DEGs in KD were enriched in systemic lupus erythematosus, suggesting a strong interplay between immunological and genetic factors in the pathogenesis of KD. These findings could significantly aid in identifying therapeutic targets and understanding KD biosignatures to design a biomarker panel for early diagnosis and severity of the disease.

## Introduction

Kawasaki Disease (KD) is an acute childhood febrile illness predominately affecting children below 5 years. It is a systemic vasculitis with a special predilection for coronary arteries (Newburger et al., 2004). It is now recognized as the most common cause of acquired heart disease in children in the developed world (Banday et al., 2021). The incidence of the KD ranges from 10 to 25 per 100,000 children below five years in North America and Europe. The highest incidence of KD is reported in children of Japanese ancestry; 350 per 100,000 children below 5 (Singh et al., 2015). Approximately 1% of all Japanese children would develop KD by the age of 10. Although many centers in India are now diagnosing children with KD on a regular basis, the vast majority of patients are perhaps still going undiagnosed and unreported (Singh et al., 2015). KD has the potential to cause severe complications and significant morbidity and mortality. We have reported that the mortality rate in our cohort has been 0.8%. This is significantly higher than mortality figures of ≤0.04% reported in developed countries (Singh et al., 2016).

Even after more than five decades of the initial description of the disease, the diagnosis of KD is still clinical and based on a constellation of clinical features. There is no pathognomonic laboratory feature. Differential diagnosis of KD includes several febrile illnesses in children, including viral infections (e.g., measles, Epstein Barr virus, and adenovirus), scarlet fever, toxic shock syndrome, and drug reactions like Stevens-Johnson syndrome or serum sickness. Clinical symptoms of KD include persistent fever for more than five days, rash, swelling of the dorsum of hands and feet, red strawberry tongue, conjunctival nonsuppurative injection, cervical lymphadenopathy, periungual peeling, and diffuse mucosal inflammation (McCrindle et al., 2017; CDC, 2020). Children with scarlet fever do not have lip changes and eye changes, which are present in KD. While rash in scarlet fever is characteristically sandpaper, KD can have a pleomorphic rash. Elevated antistreptolysin O (ASO) titer, positive throat culture for β hemolytic streptococcal group A, and brisk response to antimicrobials are other characteristic features of scarlet fever. Similarly, one can differentiate KD from other common differentials based on the constellation of clinical features (Jindal et al., 2019). Standard of care involves high-dose of intravenous immunoglobulin (IVIG) along with oral aspirin in the acute phase of the disease, which helps to resolve inflammation and minimize the risk of coronary arteries abnormalities (CAAs) (Oates-Whitehead et al., 2003). Approximately 10%–20% of children with KD doses did not respond to first-line therapy and persisted in having inflammation, known as IVIG resistant KD. Despite timely treatment, 3%–5% of patients with KD can develop CAAs. In patients having IVIG resistant KD, the chances of development of CAAs remain high (Ogata et al., 2013; Skochko et al., 2018; Brogan et al., 2020). KD remains the most common cause of acquired heart disease among children in Japan, North America, and Europe (Banday et al., 2021).

Multiple theories regarding the etiology of KD have been hypothesized such as infectious theory, infectious plus autoimmunity, RNA virus theory, and superantigen theory. However, none of them has been independently able to provide an exact mechanism for the initiation and progression of pathogenic mechanisms in KD (Ramphul and Mejias, 2018; Nakamura et al., 2019). The present consensus strongly indicates a complex interplay of infectious triggers in children with a genetic predisposition of KD, followed by an abnormal immune response (Dietz et al., 2017; Nakamura et al., 2019).

Over the last few years, high-throughput platforms such as microarray have emerged as potent tools to detect differential gene expression profiles and have been recognized as significant clinical approaches with efficient diagnosis at the molecular level, prognostic prediction, stratification of patients, and identification of therapeutic targets. To date, quite a number of genes have been examined in KD, which were usually selected based on information regarding their function in inflammation, immune response, and other biological mechanisms. Recently, gene expression profiling studies have been conducted to understand the pathophysiology of KD (Hoang et al., 2014; Dietz et al., 2017; Wu et al., 2019; Rahmati et al., 2020; Gao et al., 2021); however, the prognostic power of identified genes still remains unclear. Therefore, understanding the pathogenesis of KD is essential for identifying novel pathways that can be targeted for therapy. The current study was conducted to find out the most important hub genes among the known ones.

In our recent study, we explored the role of epigenetic factors in modulating the gene expression in KD (Sharma et al., 2021). Transcriptomic profiles in KD can reveal perturbations caused by inflammation or infection, which could help to delineate the pathogenesis of KD. In the present study, we extracted a large-scale expression dataset of a similar microarray-based platform to investigate the pathogenesis of KD. Using an integrated transcriptomic approach, we identified differentially expressed genes (DEGs) in KD. The underlying pathways were recognized using functional annotation and *via* constructing protein–protein interaction (PPI) network. This data from the present study could help to conceptualize the molecular events underpinning KD and identify diagnostic biosignature and design a biomarker panel.

## Materials and Methods

### Microarray Data

Data of microarray samples with suitable gene expression was obtained from the NCBI Gene Expression Omnibus (GEO) database (http://www.ncbi.nlm.nih.gov/geo/) (Edgar et al., 2002). Using the keywords “Kawasaki Disease and *Homo sapiens*,” a thorough search of the GEO database was performed. Furthermore, the ARGEOS web tool (https://ar-geos.org) was also used for the selection of datasets from various public databases. After a systematic review, we included GSE73461, GSE63881, GSE73463, and GSE68004, GEO datasets that belong to the same array platform GPL10558. The PRISMA checklist was followed for this study (Supplementary Figure S1).

### Differentially Expressed Genes Screening

For microarray expression data analysis ExAtlas meta-analysis software was used (Sharov et al., 2015). The four GEO datasets included in the present study were extracted from the GEO database. Data normalization was carried out using the quantile method. From the sample file, unpaired samples were removed, followed by the generation of the gene expression matrix file. Using correlation of log10-transformed expression level with other data, the quality of individual samples and level of global standard deviation were evaluated with respect to a set of pre-selected housekeeping genes. Low-quality samples were removed from the datasets.

For statistical analysis, ExAtlas uses the algorithm applied in the NIA Array Analysis (Zhou et al., 2019). The significance of gene expression change was assessed by false discovery rate (FDR) instead of *p*-values.

### Standard Meta-Analysis

In the pairwise comparison section of ExAtlas, one of the KD expression profiles was added as a sample for examination, and for baseline control, its adjacent comparative pair was added. Then, more gene expression profile pairs were added using the meta-analysis section. Furthermore, to perform the meta-analysis, a random-effect model was used to take into account the variance of heterogeneity between the studies. For each, gene symbol analysis was performed, and their effect was represented in terms of combined log-10 ratios and fold changes. FDR <0.05, *p*-value ≤0.05 and change of ≥2-folds in gene expression were considered significant.

### Protein–Protein Interaction Network Construction and Hub Gene Identification

Protein–protein interaction (PPI) network analysis was performed using the Search Tool for the Retrieval of Interacting Genes (STRING) database (https://string-db.org/). To assess possible PPI correlations, previously identified DEGs were mapped to the STRING database, followed by the extraction of PPI pairs. Cytoscape software v3.9.0 (https://cytoscape.org/) with the CytoHubba plugin was then employed to visualize the PPI network. In our study, the top 15 genes were considered as hub genes.

### Pathway Enrichment Analysis

The biological processes that are involved with the DEGs, along with the functional enrichment analysis, were studied using the BINGO app (Maere et al., 2005) of Cytoscape. A hypergeometric test was carried out using Benjamini and Hochberg FDR correction. The Gene Ontology (GO) biological process was selected as the ontology file for executing enrichment analyses. Furthermore, the Kyoto Encyclopedia of Genes and Genomes (KEGG) pathway (Kanehisa and Goto, 2000) enrichment analysis was performed using the online tool Database for Annotation, Visualization, and Integrated Discovery (DAVID) (Version 6.8; https://david.ncifcrf.gov/home.jsp) (Huang et al., 2009).

## Results

A systematic search of the studies was carried out up to December 2021. The search with the keywords “Kawasaki Disease” in *Homo sapiens* and “expression profiling by array” resulted in 13 microarray gene expression datasets (Table 1). We have combined those studies which implement the same methodology/platform and where >2 datasets were available. From 13 data sets, we have chosen four datasets which belong to the same platform GPL10558 (Table 2).

**TABLE 1:** Search result GEO datasets using the ARGEOS web tool.

Accession	Organism	Type	Platform
GSE73461	*Homo sapiens*	Expression profiling by array	GPL10558
GSE73463	*Homo sapiens*	Expression profiling by array	GPL10558
GSE63881	*Homo sapiens*	Expression profiling by array	GPL10558
GSE68004	*Homo sapiens*	Expression profiling by array	GPL10558
GSE73462	*Homo sapiens*	Expression profiling by array	GPL6947
GSE73464	*Homo sapiens*	Expression profiling by array	GPL6947
GSE48498	*Homo sapiens*	Expression profiling by array	GPL570
GSE16797	*Homo sapiens*	Expression profiling by array	GPL570
GSE9864	*Homo sapiens*	Expression profiling by array	GPL6270
GSE9863	*Homo sapiens*	Expression profiling by array	GPL6271
GSE18606	*Homo sapiens*	Expression profiling by array	GPL6480
GSE109351	*Homo sapiens*	Expression profiling by array	GPL17586
GSE73577	*Homo sapiens*	Expression profiling by array	GPL4133

**TABLE 2:** List of the datasets included in the study.

Accession	Samples	Type	Summary	References
GSE73461	459	Illumina HumanHT-12 V4.0 expression beadchip Platform: GPL10558	Genome-wide analysis of transcriptional profiles in children <17 years of age with inflammatory diseases, bacterial or viral infections, or with clinical features suggestive of an infection	Wright et al. 2018
GSE73463	233			
GSE63881	341		Transcriptional profiles in KD patients at acute and convalescent phases with different clinical outcomes were investigated	Hoang et al., 2014
GSE68004	162		1) To define the transcriptional signature of KD that can aid in the diagnosis of complete and incomplete KD in children; 2) to identify specific biomarkers that objectively discriminate between KD and other mimicking conditions, including HAdV and 3) to test the prognostic utility of GEP to determine response to IVIG therapy and development of coronary artery abnormalities (CAAs)	Jaggi et al., 2018

### Meta-Analysis

The meta-analysis identified overall 74 differentially over-expressed and 52 repressed genes in KD compared to the matched adjacent control samples. A list of the top 25 upregulated genes and 25 downregulated genes is given in Tables 3 and 4.

**TABLE 3:** Top 25 upregulated genes of the microarray meta-analyses along with their fold change values.

Upregulated genes				
Gene symbol	Gene name	Log ratio combined	Fold change combined	FDR
PPM1A	Protein phosphatase, Mg^2+^/Mn^2+^ dependent 1A	1.5612	36.408	0.001274
KIR2DL5A	Killer cell immunoglobulin-like receptor, two domains, long cytoplasmic tail, 5A	1.5063	32.085	0.004013
PPFIBP1	PTPRF interacting protein, binding protein 1 (liprin beta 1)	1.4856	30.594	0.000258
MBD3L5	Methyl-CpG binding domain protein 3-like 5	1.4677	29.354	0.0185
OSGIN1	Oxidative stress–induced growth inhibitor 1	1.3628	23.057	0.0319
FCAR	Fc fragment of IgA receptor	1.0824	12.089	0.000323
MCEMP1	Mast cell-expressed membrane protein 1	1.0476	11.158	0
HP	Haptoglobin	1.0464	11.127	0
FAM177A1	Family with sequence similarity 177 member A1	1.0097	10.227	0.000461
ANXA3	Annexin A3	1.0074	10.171	0
KCNJ15	Potassium channel, inwardly rectifying subfamily J, member 15	0.9882	9.731	5.19E-13
CEP170	Centrosomal protein 170 kDa	0.896	7.87	0.0192
FCGR1A	Fc fragment of IgG, high-affinity Ia, receptor (CD64)	0.8823	7.625	0
HMX3	H6 family homeobox 3	0.8658	7.342	0
SOD2	Superoxide dismutase 2, mitochondrial	0.8651	7.33	2.84E-06
S100A12	S100 calcium-binding protein A12	0.8571	7.196	0
H2AC20	Histone cluster 2, H2ac	0.8223	6.642	0
LIMK2	LIM domain kinase 2	0.8072	6.415	0.0275
C19orf38	Chromosome 19 open reading frame 38	0.7805	6.033	0.0396
RBMS1	RNA binding motif, single-stranded interacting protein 1	0.7598	5.751	0.00032
CYSTM1	Cysteine-rich transmembrane module containing 1	0.7549	5.687	0
ALPL	Alkaline phosphatase, liver/bone/kidney	0.7503	5.627	0
IL1R2	Interleukin 1 receptor, type II	0.7208	5.258	0
C1QB	Complement component 1, q subcomponent, B chain	0.7202	5.251	0
CCPG1	Cell cycle progression 1	0.6979	4.988	0.0239

**TABLE 4:** Top 25 downregulated genes of the microarray meta-analyses along with their fold change values.

Downregulated genes				
Gene symbol	Gene name	Log ratio combined	Fold change combined	FDR
RPL17P43	Ribosomal protein L17 pseudogene 43	−2.1741	149.314	7.33E-05
LRRC4B	Leucine-rich repeat-containing 4B	−1.9794	95.367	0.00244
OXNAD1	Oxidoreductase NAD-binding domain containing 1	−1.8402	69.215	0.006463
NR1I2	Nuclear receptor subfamily 1 group I member 2	−1.6318	42.835	0.008047
ASS1P13	Argininosuccinate synthetase 1 pseudogene 13	−1.2125	16.312	0.0476
GNLY	Granulysin	−0.9048	8.032	0.003075
SAMD3	Sterile alpha motif domain containing 3	−0.8114	6.477	0.006021
ITM2C	Integral membrane protein 2C	−0.7768	5.981	0.008414
SIRPG	Signal-regulatory protein gamma	−0.7626	5.789	0.00083
PYHIN1	Pyrin and HIN domain family member 1	−0.7397	5.492	0.0246
IL7R	Interleukin 7 receptor	−0.6883	4.879	0.000688
TLE5	Amino-terminal enhancer of split	−0.594	3.927	0.000246
TMEM204	Transmembrane protein 204	−0.5689	3.706	0.0214
TXNDC5	Thioredoxin domain containing 5 (endoplasmic reticulum)	−0.5602	3.633	0.0247
GNLY	Granulysin	−0.5543	3.583	1.47E-10
FAM83A-AS1	FAM83A antisense RNA 1	−0.5483	3.534	3.61E-06
RNF213	Ring finger protein 213	−0.5095	3.232	6.75E-05
SLAMF6	SLAM family member 6	−0.5078	3.219	0.0473
WASH3P	WAS protein family homolog 3 pseudogene	−0.488	3.076	0.0071
ADGRG1	Adhesion G protein-coupled receptor G1	−0.4751	2.986	1.35E-09
GZMK	Granzyme K	−0.4613	2.893	3.27E-07
POLR3GL	Polymerase (RNA) III (DNA directed) polypeptide G (32kD)-like	−0.4545	2.848	2.25E-05
SERPINA13P	Serpin peptidase inhibitor, clade A (alpha-1 antiproteinase, antitrypsin), member 13, pseudogene	−0.4539	2.844	0.000964
IL32	Interleukin 32	−0.4476	2.803	1.8E-06
RUNDC3A	RUN domain containing 3A	−0.4169	2.612	0.0124

### Protein–Protein Interaction Network

**FIGURE 1:**
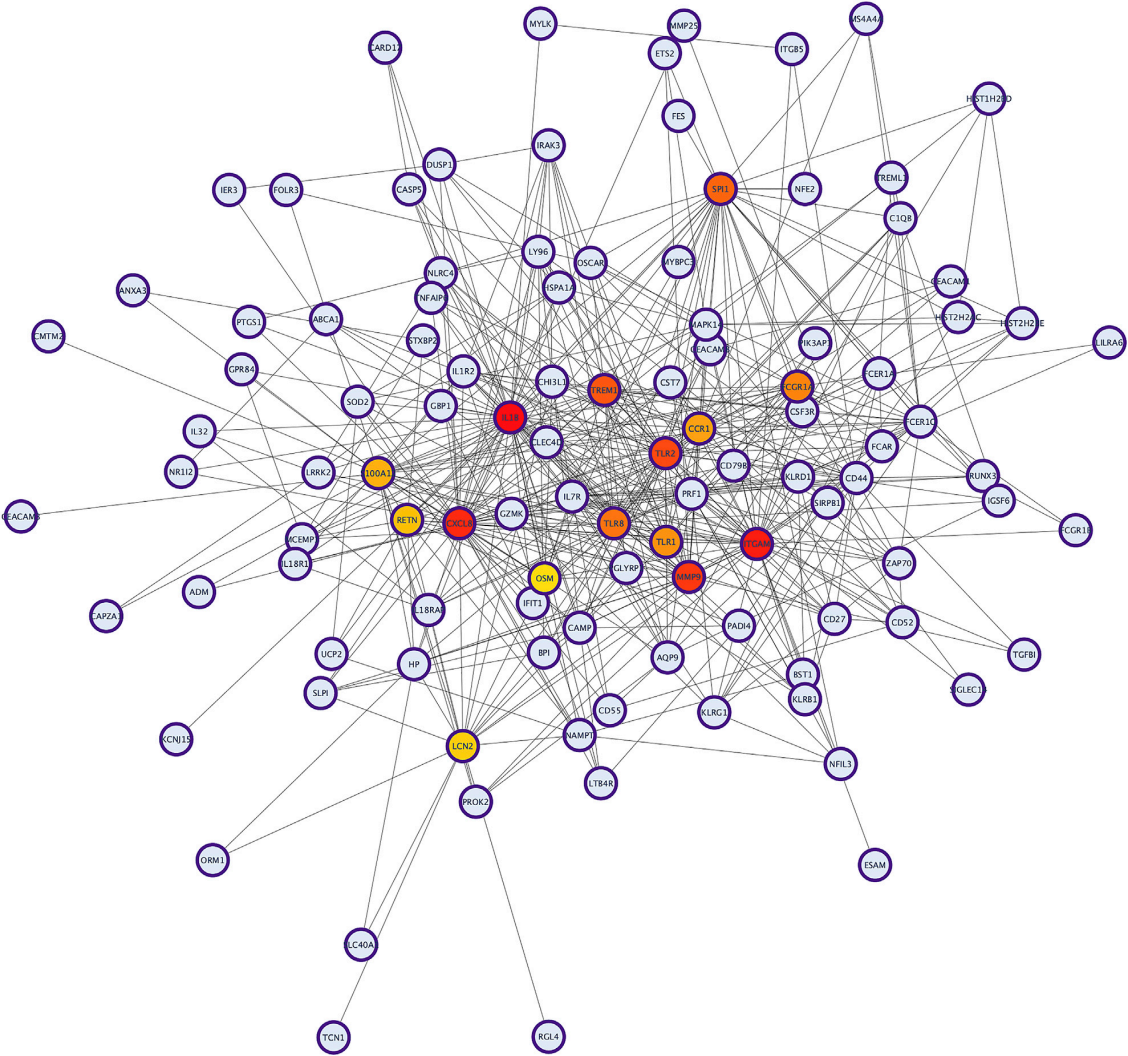
The protein–protein interaction (PPI) network analysis of differentially expressed genes in KD. The network was constructed by Cytoscape based on the PPI correlations from the STRING database.

The PPI network analysis was performed to look for the physical and functional links of proteins encoded by the identified DEGs in KD. A confidence score cut-off >0.9 was considered to construct the PPI network (Figure 1).

To identify hub proteins in the PPI network, the degree of each node in the network was calculated by using the Network Analyzer tool of Cytoscape software. The degree of a node is defined as the number of edges connected to the node. Nodes with higher degrees play a crucial role in the organization of the PPI network, and therefore they might be more crucial and relevant than non-hub genes (Vallabhajosyula et al., 2009). In this study, nodes with degrees >15 are considered to indicate “hub proteins” and are presented in Table 5. Interleukin 1-Beta (*IL1β*) had the highest degree of node (54), followed by Integrin αM (*ITGAM*) and Toll-like receptor-2 (*TLR2*), with their degrees of nodes being 44 and 42, respectively.

**TABLE 5:** Top 15 hub genes with high degrees of connectivity in patients with KD.

Genes	Betweenness centrality	Closeness centrality	Degree
IL1B	0.19793249	0.43856333	54
ITGAM	0.08124394	0.40347826	44
TLR2	0.10349151	0.41281139	42
CXCL8	0.07645807	0.40773286	42
SPI1	0.08767911	0.38538206	34
S100A12	0.07193145	0.36535433	29
MMP9	0.0400542	0.38410596	27
PRF1	0.05125956	0.37001595	27
TLR8	0.01113809	0.36535433	26
TREM1	0.01784325	0.35474006	25
CD44	0.08380828	0.38157895	25
UBB	0.10610165	0.33237822	24
FCER1G	0.01477434	0.33819242	23
IL7R	0.01549726	0.3625	23
FCGR1A	0.01234507	0.35474006	21

### Pathway Enrichment Analyses

**FIGURE 2:**
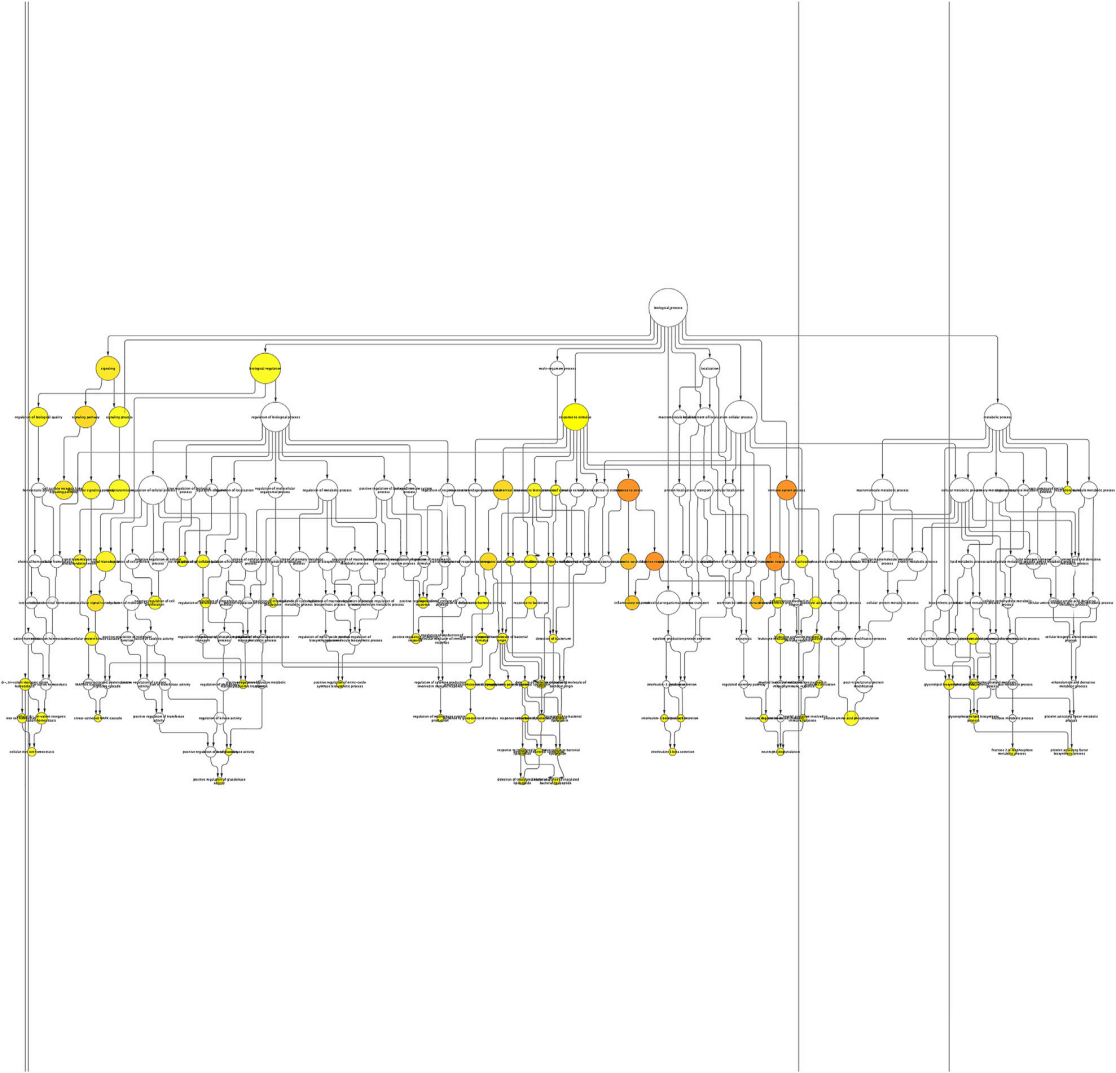
Enrichment network of the shared DEGs based on biological processes. Biological process network of differentially expressed genes of KD patients using the BINGO app of Cytoscape. Large nodes indicate more genes involved, and the size of a node is proportional to the number of targets in the GO category. Yellow nodes indicate the genes playing a significant role in KD: *p*-value < 0.05.

The BINGO plugin of Cytoscape was used to perform Gene Ontology (GO) functional enrichment analyses (Figure 2). Yellow nodes are significantly over-represented, while the white nodes are not significantly over-represented and are included only to show the yellow nodes in reference to the GO hierarchy. The size of a node is proportional to the number of query genes that are annotated to the corresponding GO category. The top 20 GO categories based on their respective node sizes, which are significantly over-represented in our study, are listed in Table 6. Among these significantly over-represented categories, the highest node size was reported for the biological regulation pathway followed by a response to stimulus and signaling. It is interesting to find that 25 genes (*ANXA3, BPI, C1QB, CCR1, CD55, CLEC4D, CXCL8, FCGR1A, IL18R1, IL18RAP, IL1B, IL32, LCN2, LILRA6, LTB4R, LY96, PGLYRP1, PRDX2, RAB27A, SH2D1A, TLR1, TLR2, TLR8, TREML1, and TRIM25*) were found to be common in top 5 pathways. This shows the importance of these genes in the flow of information in reference to the pathophysiology of the diseases. Of these 25 genes, five genes are common in between hub genes; *CXCL8, FCGR1A, IL1B, TLR2,* and *TLR8*.

**TABLE 6:** List of top 20 significantly overrepresented GO categories derived from the BINGO analysis output, based on our data. The list has been arranged in descending order of node size.

Name	Description GO	Average shortest path length	Betweenness centrality	Closeness centrality	Node size	Adjusted *p* value	No. of genes
65,007	Biological regulation	4.07075472	0.41100745	0.24565469	23.7486842	0.0394	43
50,896	Response to stimulus	4.27830189	0.24678813	0.2337376	20.4939015	0.0000	40
23,052	Signaling	4.65566038	0.02528647	0.2147923	17.8885438	0.0043	50
6,950	Response to stress	5.17924528	0.03215335	0.19307832	16.3707055	0.0000	67
23,033	Signaling pathway	5.52358491	0.01811721	0.18104184	15.7480157	0.0009	105
23,060	Signal transmission	5.16509434	0.02117959	0.19360731	14.832397	0.0314	28
23,046	Signaling process	4.58018868	0.02435299	0.21833162	14.832397	0.0314	20
7,165	Signal transduction	5.52358491	0.02548615	0.18104184	14.4222051	0.0164	34
2,376	Immune system process	4.55660377	0.07892457	0.2194617	14.1421356	0.0000	62
42,221	Response to chemical stimulus	5.14622642	0.01607496	0.19431714	13.8564065	0.0009	48
65,008	Regulation of biological quality	4.95283019	0.03262659	0.20190476	13.4164079	0.0162	32
6,955	Immune response	5.0990566	0.03425987	0.19611471	13.114877	0.0000	42
7,166	Cell surface receptor linked signaling pathway	6.51886792	0	0.15340087	12.9614814	0.0031	80
6,952	Defense response	6.0754717	0.00547133	0.16459627	12.6491106	0.0000	37
23,034	Intracellular signaling pathway	6.10377358	0.00418892	0.16383308	12.1655251	0.0132	45
10,033	Response to organic substance	5.94811321	0.00434888	0.16812054	11.6619038	0.0009	52
35,556	Intracellular signal transduction	6.33490566	0.01587904	0.15785555	11.3137085	0.0019	55
9,611	Response to wounding	6.16509434	0.00429051	0.16220352	10.5830052	0.0000	55
6,468	Protein amino acid phosphorylation	8.49528302	3.87E-04	0.11771238	9.38083152	0.0438	141
6,954	Inflammatory response	7.06132075	4.47E-05	0.14161657	8.94427191	0.0001	22

### Gene Set Overlap for Upregulated Genes

**FIGURE 3:**
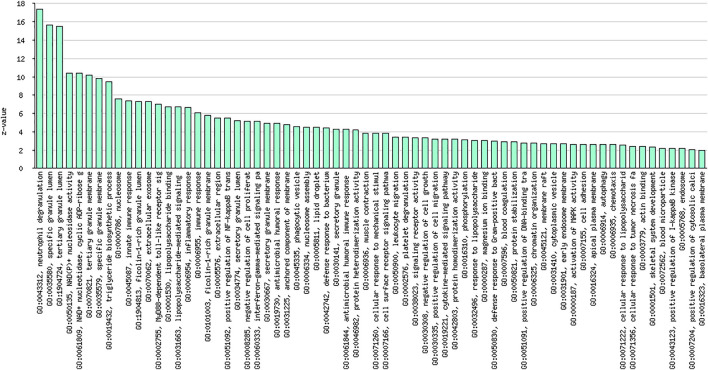
Graphical representation of significantly overrepresented individual categories of GO molecular function for the upregulated genes.

As shown in Figure 3 and Table 7, the analysis identified significantly overrepresented categories (FDR<0.05) of GO molecular function for the upregulated genes. These include “neutrophil degranulation,” “tertiary granule membrane,” and “IgG binding.” Among them, “neutrophil degranulation” and “tertiary granule membrane” were the most significantly enriched biological processes (Figure 3), while most of the proteins encoded by the upregulated genes (number = 50) were found to be located in the “neutrophil degranulation” (Table 7).

**TABLE 7:** Significantly enriched GO Molecular functions of DEGs.

Title	z-value	FDR	Fold enrichment	N genes	Genes
GO: 0043312, neutrophil degranulation	17.3639	0	7.6743	50	STXBP2, FCAR, CRISPLD2, LILRA3, RETN, S100P, CDA, TCN1, ITGAM, BST1, CAMP, OSCAR, PGLYRP1, CD55, MMP9, CEACAM8, LCN2, SLPI, CLEC4D, CYSTM1, FOLR3, MCEMP1, MMP25, QSOX1, RAB27A, VNN1, PGM2, GPR84, FTH1, CKAP4, S100A11, MAPK14, S100A12, ORM1, SIGLEC14, CANT1, TLR2, HP, QPCT, SIRPB1, CEACAM3, TNFAIP6, BPI, HSPA1A, FCER1G, CEACAM1, GYG1, ANXA3, RAB31, and CD44
GO: 0035580, specific granule lumen	15.6351	0	17.9727	15	ORM1, CANT1, HP, QPCT, BPI, RETN, TCN1, CAMP, OSCAR, PGLYRP1, LCN2, SLPI, FOLR3, QSOX1, and RAB27A
GO: 1904724, tertiary granule lumen	15.536	0	18.9095	14	MMP9, PGLYRP1, OSCAR, QSOX1, FOLR3, CDA, CAMP, TCN1, TNFAIP6, ORM1, FTH1, QPCT, HP, and CANT1

### Kyoto Encyclopedia of Genes and Genomes Pathway Analysis

KEGG pathway analysis indicated that the majority of the DEGs in KD was enriched in systemic lupus erythematosus (SLE) (Table 8).

**TABLE 8:** Significantly enriched KEGG pathway of DEGs.

Title	z-value	FDR	Fold enrichment	N genes	Genes
Systemic lupus erythematosus	4.758	0	3.9336	10	H2AC21, HLA-DRB5, H2BC18, C1QB, H2AC20, FCGR1A, H2BC21, H2AC18, H2BC5, and H3C4

## Discussion

Despite recent advancements in clinical research, the exact etiology of KD is still poorly understood, and there is no laboratory test for confirmation of diagnosis, especially when the clinical presentation is incomplete or atypical. As a result, early diagnosis of KD may be different to establish. Growing evidence from the immunological, molecular, genetic, and epigenetic studies reveal that epidemiology might differ based on the infectious trigger for KD, especially in some children with a predilection based on genetic factors (Agarwal and Agrawal, 2017; McCrindle et al., 2017; Noval Rivas and Arditi, 2020; Sharma et al., 2021). In such individuals, a putative primary immune response may be activated in mucosal lymphoid tissues. This is then followed by activation of a cytokine cascade resulting in an uncontrolled systemic immune response. Inflammation of medium-sized vessels (especially the coronary arteries) probably results from the extravasation of immune cells in the sub-endothelium. Involvement of coronary arteries results in the development of coronary artery abnormalities (CAAs) (Senzaki, 2006). Therefore, early diagnosis of KD is essential for management decisions in children with KD.

In the last decade, several studies have been conducted to identify the genetic markers for KD; however, a consistent molecular marker having a significant prognostic value has not yet been determined. Furthermore, it is known that despite treatment, approximately 3%–5% of patients with KD would still develop CAAs. Therefore, understanding the pathogenesis of KD is essential for identifying novel pathways that can be targeted for therapy. The current study used an integrated transcriptomic analysis approach to identify DEGs with significant expression change in KD. These molecular biomarkers may be used as a novel multifunctional biomarker panel for diagnosis and therapeutic targets in patients with KD.

Recently, several studies have been conducted on gene expression and genome-wide association in patients with KD. However, there is no consensus on specific genes or biological pathways responsible for the pathogenesis of KD (Popper et al., 2007; Burgner et al., 2009; Onouchi et al., 2012; Xie et al., 2018; Chen et al., 2020; Johnson et al., 2021). The present study aims to pin down specific genes using large-scale datasets on microarray profiles from the GEO database and with the aid of bioinformatics provide key hub genes. Genome-wide expression data in whole-blood samples of KD patients and in controls were obtained using four different GSE datasets with the same array platform, and an integrated bioinformatic analysis was performed. As a result, 126 shared DEGs were screened out and these were mainly enriched in immune and inflammatory responses. The identified 15 hub genes were all significantly upregulated in KD. After careful inspection, we found that five of the 15 hub genes, including *ITGAM, MMP9, S100A12, TLR2,* and *FCER1G,* were involved in the top GO item neutrophil degranulation.

The top 10 hub genes from the analyzed data sets in our study were *IL-1β, ITGAM, TLR2, CXCL8, SPI1, S100A12, MMP9, PRF1, TLR8,* and *TREM1.* Of these, IL-1β had the highest degree of a node (Lau et al., 2008). IL-1β is a potent proinflammatory cytokine that has been responsible for chronic inflammatory conditions such as cardiovascular disease, coronary artery lesions, and vasculopathy, relevant to the pathogenesis of KD (Lee et al., 2012). Enhanced circulating levels of IL-1β have been reported in KD patients as compared to controls (Hoang et al., 2014; Porritt et al., 2020). Upregulated IL-1β expression is associated with IVIG resistance (Weng et al., 2010; Fu et al., 20192019). Studies have shown that administration of IL-1 receptor antagonist (anakinra) in the KD mice model effectively prevents the development of coronary artery aneurysm, vasculitis, and myocarditis (Lee et al., 2012; Wakita et al., 2016). Based on the genetic and transcriptomic studies and evidence from the mice-model studies, anakinra is undergoing clinical trials (clinicaltrials.gov: NCT02179853) for the treatment of patients with KD.

The KEGG pathway analysis indicated that most DEGs were enriched in SLE and may thus be immune response-associated genes. While overlap of SLE and KD has been occasionally reported in the literature, this is extremely unusual. Both KD and SLE are immune-mediated disorders characterized by distinctive clinical features. However, some clinical findings are common to both conditions. These include fever, lymphadenopathy, arthritis or arthralgia, ocular and mucosal manifestations, rash, and multisystemic involvement. In addition, there appears to be a strong interplay between immunological and genetic factors in the pathogenesis of both KD and SLE (Guleria et al., 2020; Saez-de-Ocariz et al., 2020). *ITGAM* (Integrin αM) was one such gene with the second highest degree of nodes (44) in our analysis. *ITGAM* is associated with SLE (Han et al., 2009) and is found to be upregulated in KD patients with vasculopathy (Hom et al., 2008; Reindel et al., 2013). *ITGAM*, also known as *CD11b*, belongs to the integrin family and is involved in the regulation of neutrophils, monocyte activation, adhesion, and migration to damaged endothelium associated extracellular matrix. Moreover, studies have shown increased *ITGAM* levels in peripheral blood and upregulated protein expression in patients with KD (Reindel et al., 2013; Wu et al., 2019).

Immune system activation is one of the major pathogenic mechanisms of KD. These pathways play a crucial role in the innate immune response by recognizing pathogen-associated molecular patterns (PAMPs) of infectious agents. Huang et al. (2009) found that mRNA levels of *TLR2* and *TLR8* were significantly elevated in patients with KD as compared to controls. Furthermore, after treatment with IVIG therapy, the level of TLR mRNA expression in KD patients was decreased. Studies have shown that augmented expression of *TLR2* on CD14^+^ monocytes is observed in patients with KD and in coronary arteritis mice model, suggesting their role in immunopathogenesis (Rosenkranz et al., 2005; Lin et al., 2012; Mitsui et al., 2014). Therefore, these TLRs may be used as inflammatory biomarkers for the identification of patients with KD.

*CXCL2,* also known as interleukin-8 (*IL8*), is a member of the CXC chemokine family, known to possess tumorigenic properties. It is involved in proinflammatory activities, such as neutrophil degranulation, amelioration of adhesion molecule expression on neutrophils’ surfaces and directional migration of neutrophils (Brat et al., 2005). In the acute phase of KD, *IL-8* is overexpressed in mononuclear cells and polymorphonuclear neutrophils of patients with KD as compared to controls (Lin et al., 1992; Asano and Ogawa, 2001). Furthermore, Asano et al. showed that IL-8 protein and the neutrophil chemoattractant activity within plasma were increased in the acute phase of KD and were significantly elevated following IVIG therapy (Asano and Ogawa, 2001).

Spi-1 proto-oncogene (*SPI1*) is a protein-coding gene mainly involved in the activation, differentiation, activation, and migration of macrophages or B cells. This gene was also identified as one of the hub genes in a study by Gao et al. (2021). The role of the *SPI1* gene is yet not explored in KD.

Activation of *TREM-1* (triggering receptor expressed on myeloid cells-1), trigger receptor is found to be associated with the pathogenesis of KD. Zhao et al. have shown that serum soluble *TREM-1* protein concentrations were significantly higher in the acute phase of KD as compared to controls, indicating its involvement in vasculitis and CAAs in patients with KD (Zhao and Wang, 2016). Our results confirm that the expression of the *TREM* gene was significantly upregulated in patients with KD.

S100 calcium-binding protein A12 (S100A12) belongs to the S100 protein family. Serum SAA00A12 levels were significantly higher in patients with KD, and the levels declined significantly after treatment with IVIG (Foell et al., 2003; Abe et al., 20051950; Armaroli et al., 2019; Lech et al., 2019; Gao et al., 2021). Furthermore, expression of S100A12 increased on the surface of circulating endothelial cells of patients with KD and persisted for a longer duration in KD patients with CAAs, indicating the role of S100A12 in the development of CAAs. In addition, S100A12 stimulates monocytes to produce IL-8, which in turn induces coronary artery endothelial cell dysfunction (Armaroli et al., 2019). Li et al. have shown that IVIG therapy and S100A12 antibody had similar effects on reducing neutrophil infiltration (Lin et al., 1992). Data from these studies highlight the potential of S100A12 as an imperative biomarker for monitoring patients with KD. Despite this, detailed mechanisms through which S100A genes regulate the pathogenesis of KD have not yet been well studied. We hope that our results will enhance our understanding of the role of the S100A gene family in the pathogenesis of KD.

Matrix metalloproteinases-9 (*MMP-9*) has been implicated in the pathogenesis of several disorders, including tumor metastasis, inflammatory disorders, and atherosclerosis (Sakata et al., 2010). The role of *MMP-9* in the pathogenesis of KD has also been highlighted. Overexpression of MMPs induces degradation of the extracellular matrix. Takeshita et al. (2001) hypothesized that in patients with KD, activated neutrophils and monocytes produce *MMP-9* in large amounts, that upon migration leads to the breakdown of basement membrane that resulting in vascular damage and coronary artery inflammation. This hypothesis was validated in the mice model by Lau et al. (2008). Studies have shown that serum levels of *MMP-9* are elevated in KD patients and were found to play an important role in the development of CAAs (Takeshita et al., 2001; Chua et al., 2003; Gavin et al., 2003; Wu et al., 2019). Perforin-1 (*PRF1*) is one of the major cytolytic proteins of cytolytic granules. One of the main pathways of lymphocyte-mediated cytolysis entails the secretion onto target membranes of cytolytic granules contained in cytolytic effector lymphocytes of T-cell or NK-cell type (https://omim.org/entry/170280). This gene is related to hemophagocytic lymphohistiocytosis (HLH) but has no reports in KD. We assumed that together all these hub genes may play a key role in the pathogenesis of KD. However, future studies need to be conducted to verify this hypothesis.

## Conclusion

The integrated transcriptomic approach, along with the bioinformatic analysis used in the present study, helped to reveal deregulated molecular mechanisms explaining the underlying etiology of KD. Furthermore, using data from more than one microarray dataset and their healthy controls helped eliminate the potential influences of clinical, demographic, and environmental factors on transcriptomic analysis. The 10 hub genes may provide clues to understanding the pathogenesis of KD pathogenesis and could be used to design a biomarker panel to monitor patients with KD.

## Data Availability

The datasets presented in this study can be found in online repositories. The names of the repository/repositories and accession number(s) can be found in the article/Supplementary Material.
