# Supplementary material for: Exploration of Potential Biomarker Genes and Pathways in Kawasaki Disease: An Integrated in-Silico Approach
Source: Front Genet. 2022 May 9;13:849834. doi: 10.3389/fgene.2022.849834 (PMC9124956; doi:10.3389/fgene.2022.849834)
Supplement: Supplementary file 1 [file DataSheet1.docx]

**Identification of studies via databases and registers**

Records removed *before screening*:

Duplicate records removed (n =21 )

Records marked as ineligible (n = 3)

Records identified from:

GEO database (n = 21)

ARGEOS (n =32)

**Identification**

Records screened

(n =29)

Records excluded

(n =6)

Reports sought for retrieval

(n = 23)

Reports not retrieved

(n = 10)

**Screening**

Reports assessed for eligibility

(n =13)

Reports excluded:

Different array platforms (n = 5)

< 2 studies of similar platform (n = 4)

Studies included

(n = 4)

**Included**

.

*From:*  Page MJ, McKenzie JE, Bossuyt PM, Boutron I, Hoffmann TC, Mulrow CD, et al. The PRISMA 2020 statement: an updated guideline for reporting systematic reviews. BMJ 2021;372:n71. doi: 10.1136/bmj.n71

For more information, visit: <http://www.prisma-statement.org/>
